# Crystal structure of 1-(2,4-di­nitro­phen­yl)-3,5-diphenyl-1*H*-pyrazole

**DOI:** 10.1107/S2056989015021350

**Published:** 2015-11-14

**Authors:** Shaaban K. Mohamed, Joel T. Mague, Mehmet Akkurt, Mustafa R. Albayati, Alaa F. Mohamed

**Affiliations:** a Chemistry and Environmental Division, Manchester Metropolitan University, Manchester M1 5GD, England; b Chemistry Department, Faculty of Science, Minia University, 61519 El-Minia, Egypt; c Department of Chemistry, Tulane University, New Orleans, LA 70118, USA; d Department of Physics, Faculty of Sciences, Erciyes University, 38039 Kayseri, Turkey; e Kirkuk University, College of Science, Department of Chemistry, Kirkuk, Iraq; f National Organization for Drug Control and Research, Giza, Egypt

**Keywords:** crystal structure, pyrazoles, bio-active motifs

## Abstract

In the title mol­ecule, C_21_H_14_N_4_O_4_, the phenyl rings make dihedral angles of 39.61 (8) and 9.4 (1)°, respectively, with the central pyrazole ring. The dihedral angle between the pyrazole and di­nitro­phenyl rings is 46.95 (5)°. In the crystal, mol­ecules pack in helical stacks parallel to the *a* axis aided by weak C—H⋯O inter­actions.

## Related literature   

For the synthesis and pharmaceutical activities of pyrazole-containing compounds, see: Szabó *et al.* (2008 ▸); Tanitame *et al.* (2005 ▸); Cottineau *et al.* (2002 ▸); Mokhtar & El-Khawass (1988 ▸); Rida *et al.* (2009 ▸); Abadi *et al.* (2003 ▸); Sharma *et al.* (2014 ▸); Mykhailiuk (2015 ▸).
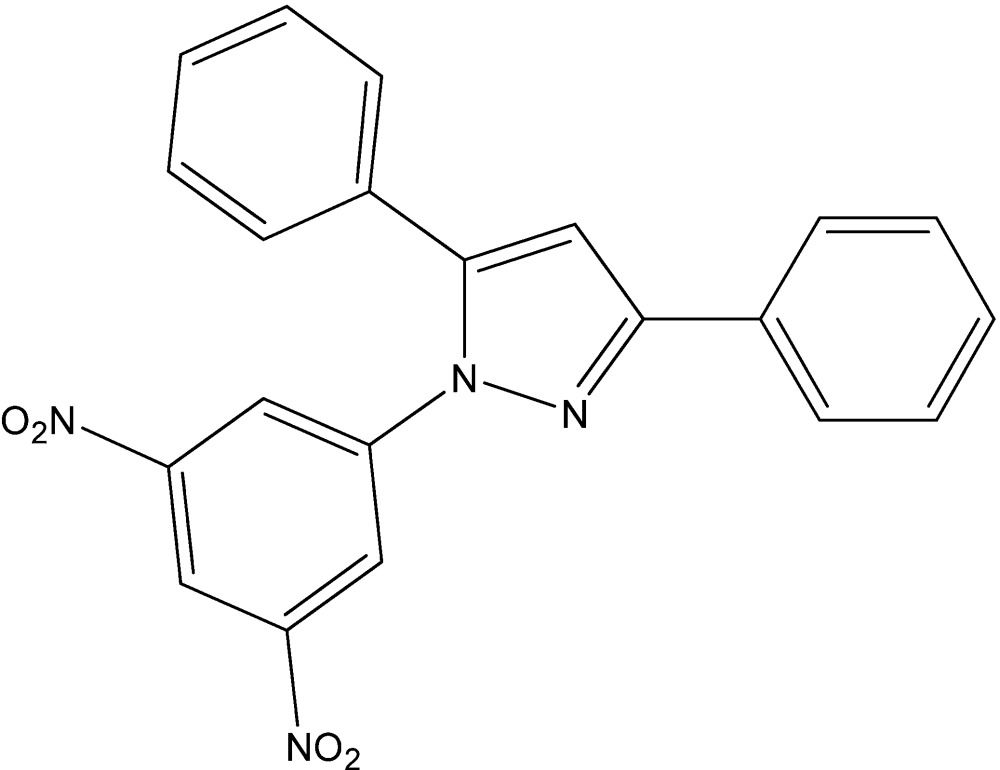


## Experimental   

### Crystal data  

C_21_H_14_N_4_O_4_
*M* *_r_* = 386.36
Orthorhombic, 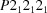

*a* = 7.2170 (5) Å
*b* = 12.9467 (10) Å
*c* = 19.3006 (14) Å
*V* = 1803.4 (2) Å^3^
*Z* = 4
Mo *K*α radiation
μ = 0.10 mm^−1^
*T* = 150 K
0.18 × 0.18 × 0.17 mm

### Data collection  

Bruker SMART APEX CCD diffractometer
Absorption correction: multi-scan (*SADABS*; Bruker, 2015 ▸) *T*_min_ = 0.78, *T*_max_ = 0.98
17283 measured reflections
4635 independent reflections
3401 reflections with *I* > 2σ(*I*)
*R*_int_ = 0.045

### Refinement  

*R*[*F* ^2^ > 2σ(*F* ^2^)] = 0.042
*wR*(*F* ^2^) = 0.096
*S* = 1.03
4635 reflections
262 parameters
H-atom parameters constrained
Δρ_max_ = 0.21 e Å^−3^
Δρ_min_ = −0.17 e Å^−3^
Absolute structure: Flack *x* determined using 1193 quotients [(*I* ^+^)−(*I* ^−^)]/[(*I* ^+^)+(*I* ^−^)] (Parsons *et al.*, 2013 ▸)
Absolute structure parameter: −0.3 (8)

### 

Data collection: *APEX2* (Bruker, 2015 ▸); cell refinement: *SAINT* (Bruker, 2015 ▸); data reduction: *SAINT*; program(s) used to solve structure: *SHELXT* (Sheldrick, 2015*a* ▸); program(s) used to refine structure: *SHELXL2014* (Sheldrick, 2015*b* ▸); molecular graphics: *DIAMOND* (Brandenburg & Putz, 2012 ▸); software used to prepare material for publication: *SHELXTL* (Sheldrick, 2008 ▸).

## Supplementary Material

Crystal structure: contains datablock(s) global, I. DOI: 10.1107/S2056989015021350/qm2114sup1.cif

Structure factors: contains datablock(s) I. DOI: 10.1107/S2056989015021350/qm2114Isup2.hkl

Supporting information file. DOI: 10.1107/S2056989015021350/qm2114Isup3.cml

**Fig. 1.**
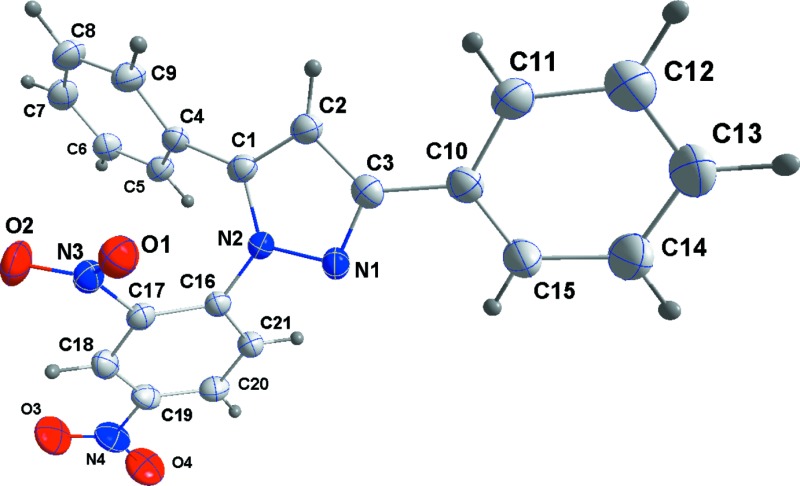
The title molecule with the labeling scheme and 50% probability ellipsoids.

**Fig. 2.**
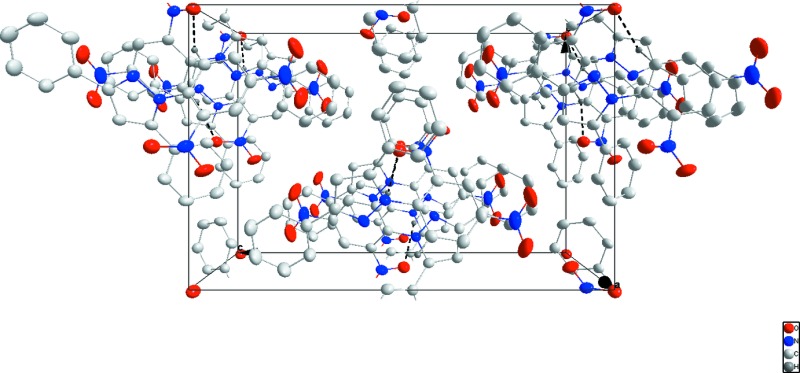
Packing viewed down the a axis with weak C—H···O interactions depicted as dotted lines.

**Fig. 3.**
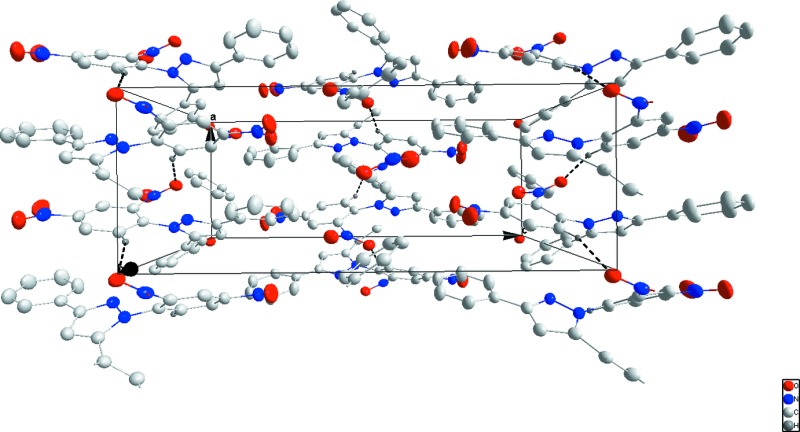
Packing viewed down the b axis with weak C—H···O interactions depicted as dotted lines.

. DOI: 10.1107/S2056989015021350/qm2114fig1.tif
The title mol­ecule with the labeling scheme and 50% probability ellipsoids.

a . DOI: 10.1107/S2056989015021350/qm2114fig2.tif
Packing viewed down the *a* axis with weak C—H⋯O inter­actions depicted as dotted lines.

b . DOI: 10.1107/S2056989015021350/qm2114fig3.tif
Packing viewed down the *b* axis with weak C—H⋯O inter­actions depicted as dotted lines.

CCDC reference: 1436135

Additional supporting information:  crystallographic information; 3D view; checkCIF report

## Figures and Tables

**Table 1:** Hydrogen-bond geometry (Å, °)

*D*—H⋯*A*	*D*—H	H⋯*A*	*D*⋯*A*	*D*—H⋯*A*
C21—H21⋯O1^i^	0.95	2.48	3.366 (3)	156

Symmetry code: (i) 
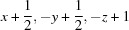
.

## supplementary crystallographic information

### S1. Comment

The heterocyclic pyrazole scaffold compounds demonstrate a remarkable wide range of pharmacological activities such as anti-inflammatory (Szabó *et al.*, 2008), anti-bacterial, antifungal (Tanitame *et al.*, 2005), hypoglycemic (Cottineau *et al.*, 2002; Mokhtar & El-Khawass, 1988), inhibition of cyclooxigenase-2 (Rida *et al.*, 2009) and anti-angiogenic (Abadi *et al.*, 2003). Different pyrazole derivatives have also shown anti-proliferative and antitumor activities (Sharma *et al*., 2014). More recently, the pyrazole ring system represents an advantageous choice for the synthesis of pharmaceutical compounds with different activities and good safety profiles (Mykhailiuk, 2015). In this context and following our on-going study of the synthesis of bio-active heterocyclic molecules we report in this study the synthesis and crystal structure of the title compound.

In the title compound (Fig. 1), the phenyl rings C4—C9 and C10—C15 make dihedral angles of 39.61 (8) and 9.4 (1)°, respectively, with the central pyrazole ring. The dihedral angle between the pyrazole and dinitrophenyl rings is 46.95 (5)°. The molecules form helical stacks running parallel to the *a* axis assisted by weak, intermolecular C21—H21···O1^i^ (i: *x* + 1/2, -*y* + 1/2, -*z* + 1) interactions (Figs. 2 and 3 and Table 1).

### S2. Experimental

An equimolar mixture of 1,3-diphenylpropane-1,3-dione (1 mmol, 224 mg) and (3,5-dinitrophenyl)hydrazine (1 mmol, 198 mg) was refluxed in 20 ml e thanol for 6–7 h. The mixture was cooled and the excess solvent was removed. The precipitate was collected and recrystallized from ethanol (m.p 421–423 K).

### S3. Refinement

H-atoms attached to carbon were placed in calculated positions (C—H = 0.95 Å). All were included as riding contributions with isotropic displacement parameters 1.2 times those of the attached atoms. The absolute structure could not be determined.

### Crystal data

**Table:**

C_21_H_14_N_4_O_4_	*D*_x_ = 1.423 Mg m^−^^3^
*M**_r_* = 386.36	Mo *K*α radiation, λ = 0.71073 Å
Orthorhombic, *P*2_1_2_1_2_1_	Cell parameters from 4944 reflections
*a* = 7.2170 (5) Å	θ = 2.6–24.1°
*b* = 12.9467 (10) Å	µ = 0.10 mm^−^^1^
*c* = 19.3006 (14) Å	*T* = 150 K
*V* = 1803.4 (2) Å^3^	Block, yellow-orange
*Z* = 4	0.18 × 0.18 × 0.17 mm
*F*(000) = 800	

### Data collection

**Table:**

Bruker SMART APEX CCD diffractometer	4635 independent reflections
Radiation source: fine-focus sealed tube	3401 reflections with *I* > 2σ(*I*)
Graphite monochromator	*R*_int_ = 0.045
Detector resolution: 8.3333 pixels mm^-1^	θ_max_ = 29.1°, θ_min_ = 1.9°
φ and ω scans	*h* = −9→9
Absorption correction: multi-scan (*SADABS*; Bruker, 2015)	*k* = −17→16
*T*_min_ = 0.78, *T*_max_ = 0.98	*l* = −26→26
17283 measured reflections	

### Refinement

**Table:**

Refinement on *F*^2^	Secondary atom site location: difference Fourier map
Least-squares matrix: full	Hydrogen site location: inferred from neighbouring sites
*R*[*F*^2^ > 2σ(*F*^2^)] = 0.042	H-atom parameters constrained
*wR*(*F*^2^) = 0.096	*w* = 1/[σ^2^(*F*_o_^2^) + (0.0375*P*)^2^] where *P* = (*F*_o_^2^ + 2*F*_c_^2^)/3
*S* = 1.03	(Δ/σ)_max_ < 0.001
4635 reflections	Δρ_max_ = 0.21 e Å^−^^3^
262 parameters	Δρ_min_ = −0.17 e Å^−^^3^
0 restraints	Absolute structure: Flack *x* determined using 1193 quotients [(*I*^+^)-(*I*^-^)]/[(*I*^+^)+(*I*^-^)] (Parsons *et al.*, 2013)
Primary atom site location: structure-invariant direct methods	Absolute structure parameter: −0.3 (8)

### Special details

**Table:**

Experimental. The diffraction data were collected in three sets of 363 frames (0.5° width in ω) at φ = 0, 120 and 240°. A scan time of 40 sec/frame was used.
Geometry. All e.s.d.'s (except the e.s.d. in the dihedral angle between two l.s. planes) are estimated using the full covariance matrix. The cell e.s.d.'s are taken into account individually in the estimation of e.s.d.'s in distances, angles and torsion angles; correlations between e.s.d.'s in cell parameters are only used when they are defined by crystal symmetry. An approximate (isotropic) treatment of cell e.s.d.'s is used for estimating e.s.d.'s involving l.s. planes.
Refinement. Refinement of *F*^2^ against ALL reflections. The weighted *R*-factor *wR* and goodness of fit *S* are based on *F*^2^, conventional *R*-factors *R* are based on *F*, with *F* set to zero for negative *F*^2^. The threshold expression of *F*^2^ > σ(*F*^2^) is used only for calculating *R*-factors(gt) *etc*. and is not relevant to the choice of reflections for refinement. *R*-factors based on *F*^2^ are statistically about twice as large as those based on *F*, and *R*- factors based on ALL data will be even larger. H-atoms attached to carbon were placed in calculated positions (C—H = 0.95 Å). All were included as riding contributions with isotropic displacement parameters 1.2 times those of the attached atoms.

### Fractional atomic coordinates and isotropic or equivalent isotropic displacement parameters (Å^2^)

**Table:**

	*x*	*y*	*z*	*U*_iso_*/*U*_eq_	
O1	0.0335 (2)	0.48865 (13)	0.50537 (9)	0.0369 (4)	
O2	0.1081 (3)	0.56459 (13)	0.40944 (10)	0.0466 (5)	
O3	0.1707 (3)	0.33782 (17)	0.21292 (9)	0.0617 (6)	
O4	0.2274 (3)	0.17545 (18)	0.22859 (9)	0.0625 (6)	
N1	0.2445 (3)	0.26495 (13)	0.58248 (9)	0.0280 (4)	
N2	0.3024 (2)	0.33719 (12)	0.53522 (9)	0.0261 (4)	
N3	0.1040 (2)	0.48950 (14)	0.44764 (10)	0.0319 (4)	
N4	0.2043 (3)	0.2633 (2)	0.24971 (11)	0.0464 (6)	
C1	0.3782 (3)	0.42265 (15)	0.56610 (11)	0.0268 (5)	
C2	0.3640 (3)	0.40575 (17)	0.63603 (11)	0.0284 (5)	
H2	0.4021	0.4511	0.6720	0.034*	
C3	0.2814 (3)	0.30750 (17)	0.64398 (11)	0.0279 (5)	
C4	0.4636 (3)	0.50690 (16)	0.52632 (11)	0.0260 (5)	
C5	0.5697 (3)	0.48621 (18)	0.46740 (12)	0.0305 (5)	
H5	0.5917	0.4167	0.4539	0.037*	
C6	0.6430 (3)	0.56641 (17)	0.42857 (12)	0.0334 (5)	
H6	0.7123	0.5519	0.3879	0.040*	
C7	0.6152 (3)	0.66760 (18)	0.44903 (13)	0.0377 (6)	
H7	0.6639	0.7226	0.4220	0.045*	
C8	0.5167 (4)	0.68884 (19)	0.50876 (13)	0.0379 (6)	
H8	0.5010	0.7583	0.5235	0.045*	
C9	0.4406 (3)	0.60891 (17)	0.54720 (12)	0.0338 (5)	
H9	0.3723	0.6239	0.5881	0.041*	
C10	0.2384 (3)	0.24957 (17)	0.70755 (11)	0.0297 (5)	
C11	0.2507 (3)	0.29519 (19)	0.77262 (12)	0.0377 (6)	
H11	0.2866	0.3656	0.7764	0.045*	
C12	0.2113 (4)	0.2394 (2)	0.83180 (13)	0.0456 (7)	
H12	0.2220	0.2715	0.8759	0.055*	
C13	0.1569 (4)	0.1382 (2)	0.82749 (13)	0.0459 (7)	
H13	0.1295	0.1004	0.8684	0.055*	
C14	0.1421 (4)	0.0917 (2)	0.76378 (14)	0.0491 (7)	
H14	0.1038	0.0217	0.7605	0.059*	
C15	0.1830 (4)	0.14687 (18)	0.70429 (13)	0.0413 (6)	
H15	0.1731	0.1139	0.6605	0.050*	
C16	0.2618 (3)	0.31836 (16)	0.46461 (10)	0.0254 (4)	
C17	0.1820 (3)	0.39223 (16)	0.42117 (11)	0.0277 (5)	
C18	0.1647 (3)	0.37515 (18)	0.35076 (12)	0.0329 (5)	
H18	0.1168	0.4271	0.3210	0.040*	
C19	0.2188 (3)	0.28098 (18)	0.32499 (11)	0.0328 (5)	
C20	0.2869 (3)	0.20328 (18)	0.36702 (12)	0.0332 (5)	
H20	0.3171	0.1376	0.3482	0.040*	
C21	0.3104 (3)	0.22280 (16)	0.43688 (11)	0.0293 (5)	
H21	0.3600	0.1707	0.4662	0.035*	

### Atomic displacement parameters (Å^2^)

**Table:**

	*U*^11^	*U*^22^	*U*^33^	*U*^12^	*U*^13^	*U*^23^
O1	0.0335 (9)	0.0348 (10)	0.0423 (10)	0.0026 (7)	0.0025 (8)	0.0002 (8)
O2	0.0523 (11)	0.0286 (9)	0.0588 (11)	0.0032 (8)	−0.0003 (10)	0.0159 (9)
O3	0.0703 (13)	0.0840 (15)	0.0308 (10)	−0.0060 (12)	−0.0092 (9)	0.0152 (10)
O4	0.0708 (14)	0.0772 (15)	0.0394 (11)	0.0100 (12)	−0.0055 (10)	−0.0219 (10)
N1	0.0297 (10)	0.0279 (10)	0.0264 (9)	−0.0008 (8)	−0.0002 (8)	0.0051 (8)
N2	0.0293 (9)	0.0228 (9)	0.0261 (9)	−0.0012 (8)	−0.0007 (7)	0.0017 (7)
N3	0.0264 (9)	0.0276 (10)	0.0418 (12)	−0.0005 (8)	−0.0052 (9)	0.0052 (9)
N4	0.0402 (12)	0.0702 (17)	0.0289 (12)	−0.0053 (12)	−0.0022 (9)	−0.0018 (12)
C1	0.0261 (11)	0.0231 (11)	0.0313 (12)	0.0014 (9)	−0.0008 (9)	−0.0022 (9)
C2	0.0300 (11)	0.0255 (11)	0.0297 (11)	−0.0004 (9)	−0.0019 (10)	−0.0021 (9)
C3	0.0276 (11)	0.0303 (12)	0.0256 (11)	0.0037 (9)	−0.0006 (9)	−0.0009 (9)
C4	0.0235 (11)	0.0260 (12)	0.0286 (11)	−0.0011 (9)	−0.0032 (9)	−0.0004 (9)
C5	0.0279 (12)	0.0264 (12)	0.0372 (13)	0.0003 (9)	−0.0004 (10)	−0.0019 (10)
C6	0.0280 (12)	0.0363 (13)	0.0360 (13)	−0.0027 (10)	0.0023 (10)	0.0017 (11)
C7	0.0347 (12)	0.0318 (13)	0.0467 (14)	−0.0085 (10)	−0.0016 (12)	0.0058 (11)
C8	0.0421 (14)	0.0253 (13)	0.0462 (15)	−0.0045 (11)	−0.0027 (11)	−0.0031 (11)
C9	0.0396 (13)	0.0282 (12)	0.0335 (12)	−0.0011 (10)	0.0005 (11)	−0.0059 (11)
C10	0.0274 (11)	0.0340 (13)	0.0278 (11)	0.0006 (10)	−0.0014 (10)	0.0005 (10)
C11	0.0420 (14)	0.0382 (14)	0.0329 (13)	−0.0083 (11)	0.0034 (11)	−0.0026 (10)
C12	0.0544 (17)	0.0531 (16)	0.0293 (13)	−0.0042 (14)	0.0032 (12)	−0.0003 (12)
C13	0.0553 (17)	0.0519 (17)	0.0304 (14)	−0.0078 (14)	0.0004 (12)	0.0116 (12)
C14	0.0721 (19)	0.0360 (15)	0.0394 (14)	−0.0103 (14)	−0.0031 (14)	0.0091 (12)
C15	0.0616 (16)	0.0329 (13)	0.0293 (12)	−0.0054 (12)	−0.0031 (12)	0.0015 (10)
C16	0.0234 (10)	0.0264 (11)	0.0265 (11)	−0.0034 (9)	−0.0013 (9)	0.0024 (9)
C17	0.0257 (11)	0.0255 (11)	0.0320 (12)	−0.0043 (9)	−0.0007 (9)	0.0029 (10)
C18	0.0300 (12)	0.0364 (13)	0.0324 (12)	−0.0055 (10)	−0.0040 (10)	0.0092 (10)
C19	0.0315 (12)	0.0430 (14)	0.0240 (11)	−0.0077 (11)	−0.0020 (10)	0.0011 (10)
C20	0.0321 (12)	0.0335 (13)	0.0340 (13)	−0.0033 (10)	0.0014 (10)	−0.0056 (10)
C21	0.0294 (12)	0.0288 (12)	0.0296 (12)	−0.0015 (9)	−0.0025 (9)	0.0022 (9)

### Geometric parameters (Å, º)

**Table:**

O1—N3	1.225 (2)	C8—C9	1.387 (3)
O2—N3	1.220 (2)	C8—H8	0.9500
O3—N4	1.222 (3)	C9—H9	0.9500
O4—N4	1.220 (3)	C10—C15	1.390 (3)
N1—C3	1.335 (3)	C10—C11	1.391 (3)
N1—N2	1.372 (2)	C11—C12	1.381 (3)
N2—C1	1.371 (3)	C11—H11	0.9500
N2—C16	1.415 (3)	C12—C13	1.371 (4)
N3—C17	1.471 (3)	C12—H12	0.9500
N4—C19	1.475 (3)	C13—C14	1.373 (4)
C1—C2	1.371 (3)	C13—H13	0.9500
C1—C4	1.469 (3)	C14—C15	1.384 (3)
C2—C3	1.413 (3)	C14—H14	0.9500
C2—H2	0.9500	C15—H15	0.9500
C3—C10	1.471 (3)	C16—C21	1.393 (3)
C4—C9	1.391 (3)	C16—C17	1.396 (3)
C4—C5	1.397 (3)	C17—C18	1.382 (3)
C5—C6	1.385 (3)	C18—C19	1.373 (3)
C5—H5	0.9500	C18—H18	0.9500
C6—C7	1.383 (3)	C19—C20	1.383 (3)
C6—H6	0.9500	C20—C21	1.382 (3)
C7—C8	1.382 (3)	C20—H20	0.9500
C7—H7	0.9500	C21—H21	0.9500
			
C3—N1—N2	104.42 (17)	C15—C10—C11	117.7 (2)
C1—N2—N1	112.51 (17)	C15—C10—C3	120.71 (19)
C1—N2—C16	129.82 (17)	C11—C10—C3	121.5 (2)
N1—N2—C16	117.39 (16)	C12—C11—C10	120.8 (2)
O2—N3—O1	124.5 (2)	C12—C11—H11	119.6
O2—N3—C17	117.58 (19)	C10—C11—H11	119.6
O1—N3—C17	117.85 (17)	C13—C12—C11	120.6 (2)
O4—N4—O3	124.7 (2)	C13—C12—H12	119.7
O4—N4—C19	117.7 (2)	C11—C12—H12	119.7
O3—N4—C19	117.6 (2)	C12—C13—C14	119.7 (2)
N2—C1—C2	105.63 (19)	C12—C13—H13	120.1
N2—C1—C4	122.64 (19)	C14—C13—H13	120.1
C2—C1—C4	131.6 (2)	C13—C14—C15	120.0 (2)
C1—C2—C3	106.38 (19)	C13—C14—H14	120.0
C1—C2—H2	126.8	C15—C14—H14	120.0
C3—C2—H2	126.8	C14—C15—C10	121.2 (2)
N1—C3—C2	111.04 (18)	C14—C15—H15	119.4
N1—C3—C10	119.27 (18)	C10—C15—H15	119.4
C2—C3—C10	129.68 (19)	C21—C16—C17	118.79 (19)
C9—C4—C5	118.9 (2)	C21—C16—N2	118.11 (18)
C9—C4—C1	120.2 (2)	C17—C16—N2	123.06 (18)
C5—C4—C1	120.87 (19)	C18—C17—C16	121.2 (2)
C6—C5—C4	120.4 (2)	C18—C17—N3	116.36 (19)
C6—C5—H5	119.8	C16—C17—N3	122.39 (18)
C4—C5—H5	119.8	C19—C18—C17	118.2 (2)
C7—C6—C5	120.0 (2)	C19—C18—H18	120.9
C7—C6—H6	120.0	C17—C18—H18	120.9
C5—C6—H6	120.0	C18—C19—C20	122.3 (2)
C8—C7—C6	120.1 (2)	C18—C19—N4	118.3 (2)
C8—C7—H7	120.0	C20—C19—N4	119.4 (2)
C6—C7—H7	120.0	C21—C20—C19	118.9 (2)
C7—C8—C9	120.1 (2)	C21—C20—H20	120.6
C7—C8—H8	120.0	C19—C20—H20	120.6
C9—C8—H8	120.0	C20—C21—C16	120.4 (2)
C8—C9—C4	120.4 (2)	C20—C21—H21	119.8
C8—C9—H9	119.8	C16—C21—H21	119.8
C4—C9—H9	119.8		
			
C3—N1—N2—C1	1.4 (2)	C11—C12—C13—C14	0.3 (4)
C3—N1—N2—C16	−173.15 (17)	C12—C13—C14—C15	0.3 (5)
N1—N2—C1—C2	−1.6 (2)	C13—C14—C15—C10	−0.3 (4)
C16—N2—C1—C2	172.1 (2)	C11—C10—C15—C14	−0.2 (4)
N1—N2—C1—C4	174.98 (19)	C3—C10—C15—C14	−179.9 (3)
C16—N2—C1—C4	−11.4 (3)	C1—N2—C16—C21	135.4 (2)
N2—C1—C2—C3	1.1 (2)	N1—N2—C16—C21	−51.2 (3)
C4—C1—C2—C3	−175.0 (2)	C1—N2—C16—C17	−42.3 (3)
N2—N1—C3—C2	−0.6 (2)	N1—N2—C16—C17	131.2 (2)
N2—N1—C3—C10	−179.24 (18)	C21—C16—C17—C18	−5.3 (3)
C1—C2—C3—N1	−0.3 (2)	N2—C16—C17—C18	172.3 (2)
C1—C2—C3—C10	178.1 (2)	C21—C16—C17—N3	171.68 (19)
N2—C1—C4—C9	142.0 (2)	N2—C16—C17—N3	−10.7 (3)
C2—C1—C4—C9	−42.4 (4)	O2—N3—C17—C18	−32.5 (3)
N2—C1—C4—C5	−38.4 (3)	O1—N3—C17—C18	144.9 (2)
C2—C1—C4—C5	137.2 (3)	O2—N3—C17—C16	150.3 (2)
C9—C4—C5—C6	−3.2 (3)	O1—N3—C17—C16	−32.3 (3)
C1—C4—C5—C6	177.2 (2)	C16—C17—C18—C19	3.6 (3)
C4—C5—C6—C7	1.6 (3)	N3—C17—C18—C19	−173.60 (19)
C5—C6—C7—C8	1.0 (4)	C17—C18—C19—C20	0.8 (3)
C6—C7—C8—C9	−2.0 (4)	C17—C18—C19—N4	−178.7 (2)
C7—C8—C9—C4	0.4 (4)	O4—N4—C19—C18	−169.8 (2)
C5—C4—C9—C8	2.2 (3)	O3—N4—C19—C18	10.5 (3)
C1—C4—C9—C8	−178.2 (2)	O4—N4—C19—C20	10.6 (3)
N1—C3—C10—C15	8.4 (3)	O3—N4—C19—C20	−169.1 (2)
C2—C3—C10—C15	−169.9 (2)	C18—C19—C20—C21	−3.4 (3)
N1—C3—C10—C11	−171.3 (2)	N4—C19—C20—C21	176.2 (2)
C2—C3—C10—C11	10.4 (4)	C19—C20—C21—C16	1.6 (3)
C15—C10—C11—C12	0.8 (4)	C17—C16—C21—C20	2.7 (3)
C3—C10—C11—C12	−179.5 (2)	N2—C16—C21—C20	−175.03 (19)
C10—C11—C12—C13	−0.8 (4)		

### Hydrogen-bond geometry (Å, º)

**Table:**

*D*—H···*A*	*D*—H	H···*A*	*D*···*A*	*D*—H···*A*
C21—H21···O1^i^	0.95	2.48	3.366 (3)	156

Symmetry code: (i) *x*+1/2, −*y*+1/2, −*z*+1.

## References

[bb1] Abadi, A. H., Eissa, A. A. H. & Hassan, E. (2003). *Chem. Pharm. Bull.* **51**, 838–844. 10.1248/cpb.51.838 12843591

[bb2] Brandenburg, K. & Putz, H. (2012). *DIAMOND*. Crystal Impact GbR, Bonn, Germany.

[bb3] Bruker (2015). *APEX2*, *SADABS* and *SAINT*. Bruker AXS Inc., Madison, Wisconsin, USA.

[bb4] Cottineau, B., Toto, P., Marot, C., Pipaud, A. & Chenault, J. (2002). *Bioorg. Med. Chem. Lett.* **12**, 2105–2108. 10.1016/s0960-894x(02)00380-3 12127514

[bb5] Mokhtar, H. M. & El-Khawass, S. M. (1988). *Jnl Chin. Chem. Soc.* **35**, 57–62.

[bb6] Mykhailiuk, P. K. (2015). *Beilstein J. Org. Chem.* **11**, 16–24. 10.3762/bjoc.11.3 PMC4311722 25670987

[bb7] Parsons, S., Flack, H. D. & Wagner, T. (2013). *Acta Cryst.* B**69**, 249–259. 10.1107/S2052519213010014 PMC3661305 23719469

[bb8] Rida, S. M., Saudi, M. N. S., Youssef, A. M. & Halim, M. A. (2009). *Lett. Org. Chem.* **6**, 282–288.

[bb9] Sharma, V., Sharma, V., Kumar, V. & Kumar, V. (2014). *Pak. J. Pharm. Sci.* **27**, 1851–1855. 25362609

[bb10] Sheldrick, G. M. (2008). *Acta Cryst.* A**64**, 112–122. 10.1107/S0108767307043930 18156677

[bb11] Sheldrick, G. M. (2015*a*). *Acta Cryst.* A**71**, 3–8.

[bb12] Sheldrick, G. M. (2015*b*). *Acta Cryst.* C**71**, 3–8.

[bb13] Szabó, G., Fischer, J., Kis-Varga, Á. & Gyires, K. (2008). *J. Med. Chem.* **51**, 142–147. 10.1021/jm070821f 18072726

[bb14] Tanitame, A., Oyamada, Y., Ofuji, K., Terauchi, H., Kawasaki, M., Wachi, M. & Yamagishi, J. (2005). *Bioorg. Med. Chem. Lett.* **15**, 4299–4303. 10.1016/j.bmcl.2005.06.103 16087337

