# Supplementary figures and images for: Crystal structure of 1-(2,4-di­nitro­phen­yl)-3,5-diphenyl-1H-pyrazole
Source: Acta Crystallogr E Crystallogr Commun. 2015 Nov 14;71(Pt 12):o931–2. doi: 10.1107/S2056989015021350 (PMC4719887; doi:10.1107/S2056989015021350)

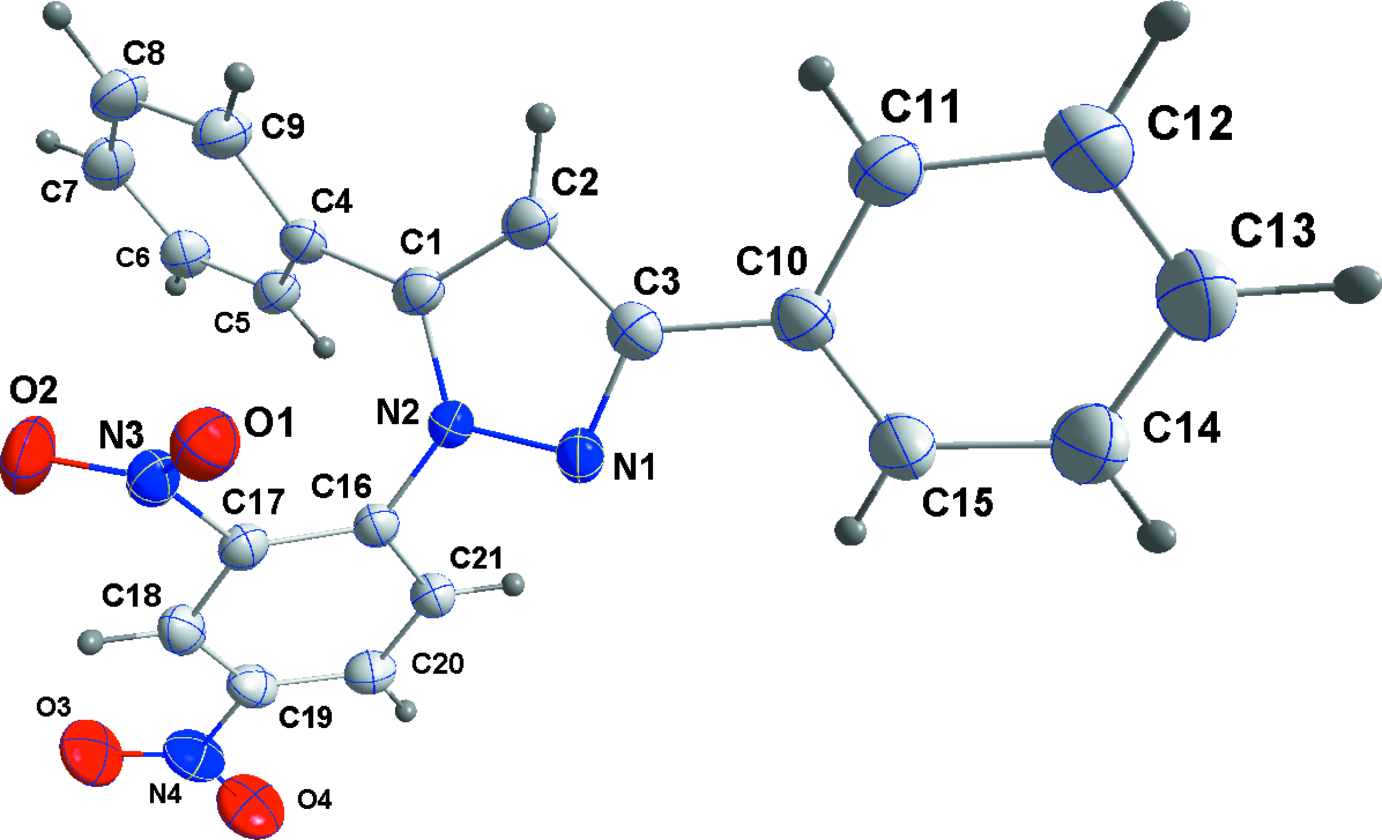

Supplement: Supplementary file 4 [file e-71-0o931-fig1.tif]

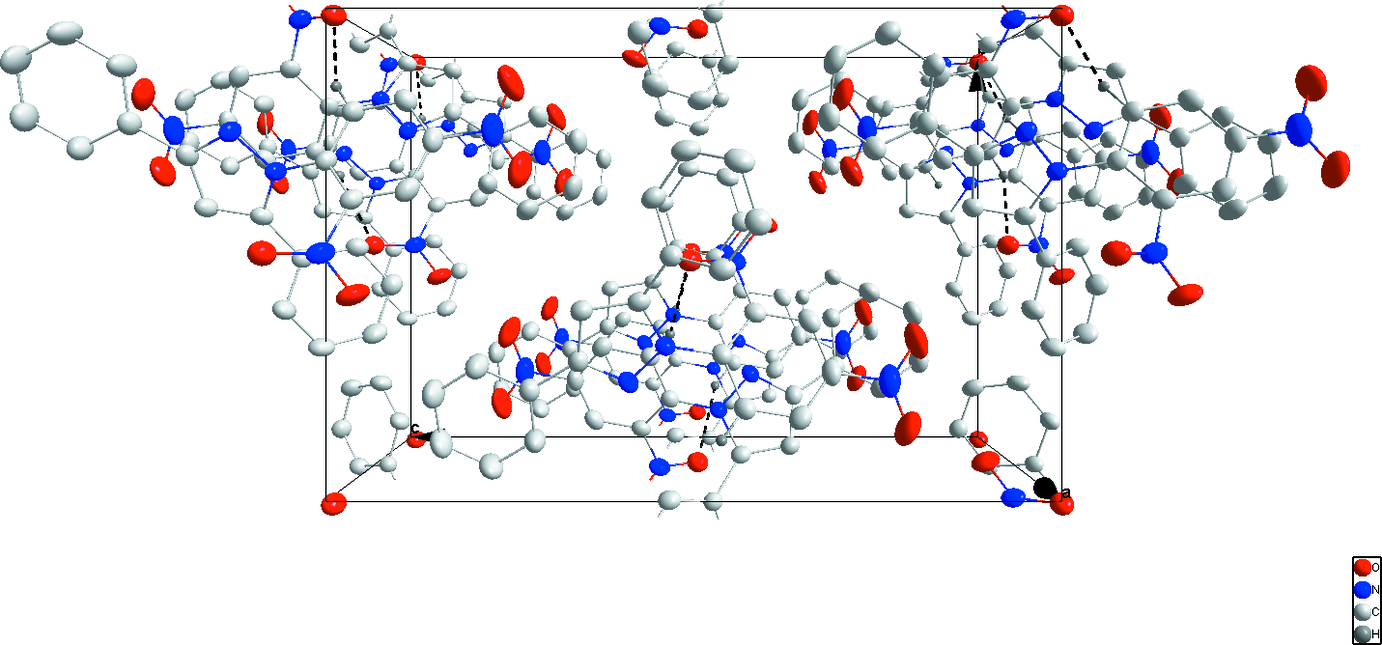

Supplement: Supplementary file 5 [file e-71-0o931-fig2.tif]

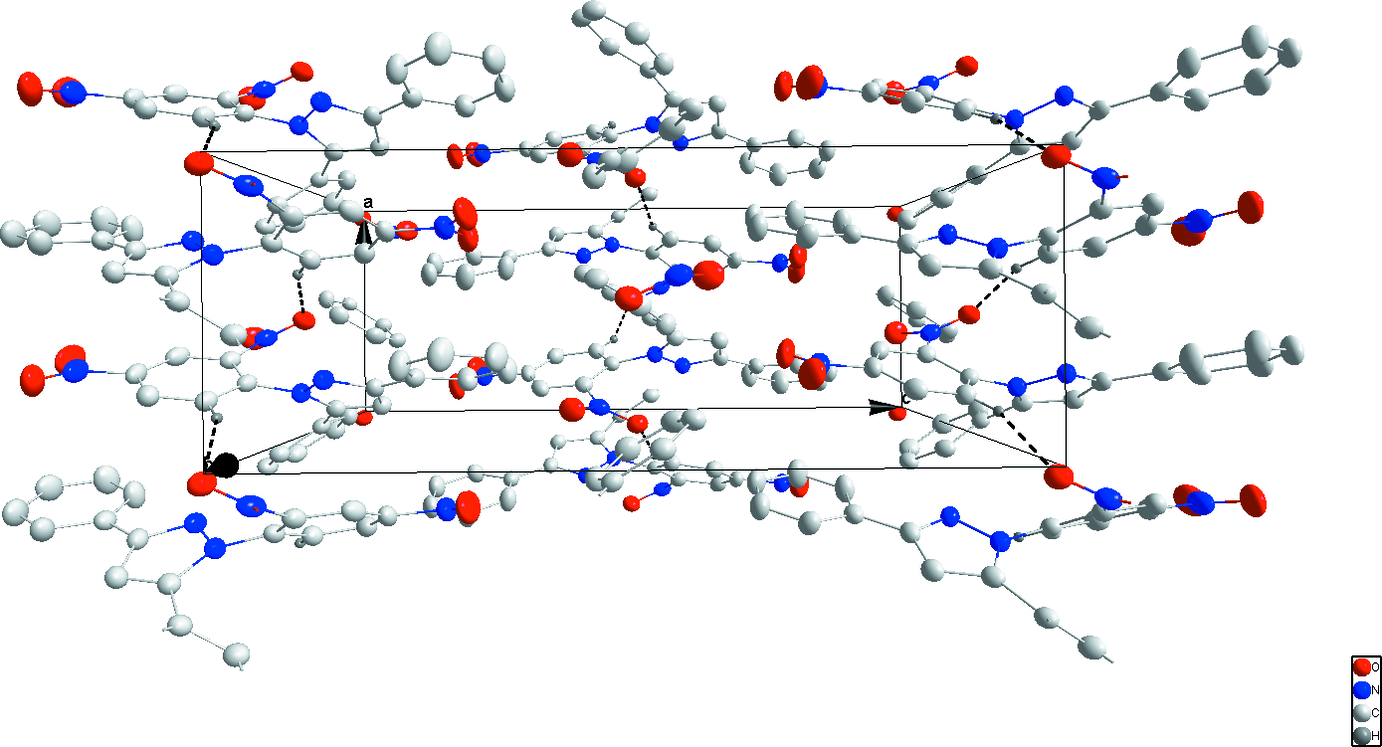

Supplement: Supplementary file 6 [file e-71-0o931-fig3.tif]
